# Sarcopenia, body composition, physical performance, and clinical features in Parkinson’s disease: a cross-sectional comparative study

**DOI:** 10.3389/fendo.2026.1793254

**Published:** 2026-03-17

**Authors:** João Rafael Gomes de Luna, Danielle Pessoa Lima, Vlademir Carneiro Gomes, Fábia Karine de Moura Lopes, Samuel Brito de Almeida, Pedro Lucas de Souza Barroso, Heitor Rios de Medeiros Virginio, Antonio Brazil Viana Júnior, Virgínia Oliveira Fernandes, Jarbas de Sá Roriz-Filho, Pedro Braga-Neto, Renan Magalhães Montenegro-Júnior

**Affiliations:** 1 Division of Geriatrics, Department of Clinical Medicine, Universidade Federal do Ceará, Fortaleza, Ceará, Brazil; 2 Clinical Research Unit, Hospital Universitário Walter Cantídio, Universidade Federal do Ceará/Empresa Brasileira de Serviços Hospitalares (EBSERH), Fortaleza, Ceará, Brazil; 3 Medical School, Universidade Federal do Ceará, Fortaleza, Ceará, Brazil; 4 Division of Endocrinology and Metabolism, Department of Clinical Medicine, Universidade Federal do Ceará, Fortaleza, Ceará, Brazil; 5 Division of Neurology, Department of Clinical Medicine, Universidade Federal do Ceará, Fortaleza, Ceará, Brazil

**Keywords:** body composition, muscle strength, Parkinson’s disease, physical performance, sarcopenia

## Abstract

**Objective:**

To compare sarcopenia prevalence, body composition, physical performance, and related clinical features between middle-aged and older patients with mild-to-moderate Parkinson’s disease (PD) and matched controls.

**Methods:**

A cross-sectional study carried out at a tertiary hospital in Brazil. Patients with PD in Hoehn and Yahr stages 1–3 and controls matched for age, sex and comorbidities were assessed. We measured handgrip strength with a hand dynamometer. Body composition and muscle mass were evaluated using DEXA. Sarcopenia was diagnosed following EWGSOP2 criteria. Clinical, cognitive and nutritional assessments were also performed.

**Results:**

The study population comprised 250 patients (124 with PD), of whom one hundred and nine (43.6%) were female. The median age was 69.5 (IQR 61.9 – 76). Similar prevalences of sarcopenia were found among PD patients (9.7%) and controls (11.1%). PD patients had higher SARC-F scores, worse balance, higher gait speed, and more frequent falls. After adjustment for age, hypertension, and type 2 diabetes, quantile regression showed a downward trend in handgrip strength and appendicular skeletal muscle mass index among PD patients, with a significant muscle mass reduction at the 40^th^ percentile. PD patients also had lower calf circumference and fat mass, despite higher protein intake.

**Conclusion:**

Patients with PD had worse balance, greater fall risk, and lower muscle mass and calf circumference compared to controls, despite similar sarcopenia prevalence. These results highlight subtle but potentially clinically relevant impairments that may not be captured by traditional mean-based analyses and categorical sarcopenia diagnosis.

## Introduction

Parkinson’s disease (PD) is a progressive neurodegenerative disease characterized by motor and non-motor symptoms that impair physical function and functionality. Its prevalence increases with aging, making it a significant public health concern (1, 2). PD has been also recently linked to increased risk of sarcopenia, which is a complex and multifactorial condition characterized by abnormally reduced muscle mass quantity and quality and also predisposes to increased morbidity and mortality (3, 4).

The PD–sarcopenia relationship remains debated. Some studies suggest bidirectionality:

Sarcopenia may increase future PD risk (5), while genetic evidence indicates protective roles for strength and lean mass against levodopa-induced dyskinesia (6). Early sarcopenia was linked to higher UPDRS-III scores (7). PD may contribute to muscle loss through neuroinflammation, oxidative damage, α-synuclein accumulation in muscle, reduced motor neuron density, hormonal dysregulation, and intestinal dysbiosis. Dopaminergic degeneration may impair cortical motor drive and motor unit recruitment, leading to inefficient muscle activation and progressive loss of strength and mass (3).

Despite the topic’s clinical relevance, literature is limited by methodological inconsistencies, such as different diagnostic criteria and lack of control for confounding factors (8, 9). In this sense, comprehensive comparative studies are needed to better assess the potential independent connections between PD and loss of muscle mass and strength. The present study aimed to compare muscle health –including sarcopenia prevalence according to the European Working Group on Sarcopenia in Older People 2 (EWGSOP2) diagnostic criteria (10), muscle mass, and muscle strength – along with physical performance, body composition, risk of falls, and related clinical features between middle-aged and older patients with mild-to-moderate PD and controls, while adjusting for major independent risk factors for sarcopenia. We hypothesized that patients with mild-to-moderate PD would exhibit clinically relevant reductions in muscle mass and strength, along with worse physical performance and higher fall risk compared with matched controls.

## Materials and methods

### Study participants

This is a cross-sectional study conducted at Hospital Universitário Walter Cantídio (HUWC), Fortaleza, Brazil, between May 2021 and May 2023. PD patients attended regularly the Movement Disorders outpatient clinic at HUWC. The diagnosis was made according to the Movement Disorder Society (MDS) criteria by two neurologists and one geriatrician (11). The following eligibility criteria were adopted in this study: modified Hoehn and Yahr (HY) stage 1-3, age ≥40 years, and ability to independently stand and walk. Patients in HY 4–5 were not included in the study as they cannot perform the Five Times Sit-to-Stand (FTSTS) test, as well as balance and gait speed tests. Also, patients with HY stage 5 are no longer followed in face-to-face consultations at the outpatient clinic, given that home care is recommended for them. The control group met the same criteria, except for not having PD. The majority of patients (n=105) were recruited from the Geriatrics outpatient clinic. The remaining attended other HUWC outpatient clinics, such as Internal Medicine, Pulmonology, and Cardiology.

Patients with severe health conditions or uncontrolled chronic diseases that could compromise their safety or impact how the data were interpreted were excluded, as follows: heart failure NYHA class III-IV; dialysis-dependent end-stage renal disease; non-PD neurological diseases with motor impairment, severe/very severe COPD, severe osteoarthritis, active malignancy (except localized prostate/skin cancer), or moderate/severe dementia (Clinical Dementia Rating 2-3). Recent administration of gastrointestinal contrast or radionuclides (<72h), pregnancy, deep brain stimulation and heart pacemaker were also exclusion criteria since they would hamper the interpretation of dual-energy X-ray absorptiometry (DEXA).

The sample size calculation was based on previously reported sarcopenia prevalence estimates of approximately 16% in national community-dwelling older adults (12) and up to 50% in patients with Parkinson’s disease (13). With a two-sided significance level of 5% and 80% statistical power, the estimated minimum sample size was 35 participants per group (1:1 allocation).

All participants provided written informed consent for the study, which was authorized by the HUWC Research Ethics Committee (approval 91075318.1.0000.5045).

### Clinical assessment

We employed an interview that was structured to collect sociodemographic and clinical data. The information was cross-checked with caregivers, relatives and clinical records. Data on antiparkinsonian medications provided by the Brazilian public health system (L-dopa, COMT inhibitors, MAO-B inhibitors, amantadine, and dopamine agonists) were collected. The conversion formulas proposed by Tomlinson et al. were used to calculate the levodopa equivalent doses (LED) (14). The modified HY scale was used to assess PD stage and the part III MDS-UPDRS for motor symptom severity. We also used the Schwab and England Activities of Daily Living (SE ADL) scale to evaluate functional status.

Patients were assessed for depressive symptoms and cognitive performance using the 15-item Geriatric Depression Scale (GDS-15) and the Mini-Mental State Examination (MMSE), respectively. Cognitive performance and depressive symptoms were assessed to account for non-motor features that may influence physical performance, functional status, and fall risk. These assessments were conducted by the same trained researchers, in a private, quiet, and air-conditioned room, without interruptions. Functional status in the control group was assessed with the Katz Index. Falls were defined as “a situation in which the patient inadvertently dropped to the ground or another lower level, not attributable to seizure, accident, or syncope”, and recorded for the past 1 and 6 months (15). Recurrent falls were defined as ≥ 2 falls in the last 12 months.

### Sarcopenia and body composition

Assessment for sarcopenia was performed according to EWGSOP2 guidelines. All patients were screened with the SARC-F questionnaire, a five-item tool that assesses perceived limitations in strength, walking ability, chair rise, stair climbing, and the occurrence of falls (16). We used the SAEHAN^®^ dynamometer to measure grip strength following the Southampton protocol: three trials per side, recording the highest value (17). Cut-off points for grip strength were 27 kg for men and 16 kg for women (10). Although the EWGSOP2 recommends both handgrip strength and the FTSTS test as measures of low muscle strength, the FTSTS test is considered a functional proxy of lower limb strength rather than a direct measure of muscle strength (10). In patients with PD, performance on this test may be substantially influenced by bradykinesia, rigidity, and balance impairment, which can confound its interpretation as a strength measure. It has been validated to assess risk of falls in this population but was not strongly correlated to lower limb strength in multiple regression analysis. Duncan et al. (18) demonstrated that, in PD, balance and bradykinesia measures explained a significant proportion of the variance (53%) in chair stand time (18). For these reasons, we prioritized handgrip strength for the operational definition of low muscle strength.

Body composition was evaluated using DEXA. The following measures were recorded: total and appendicular skeletal muscle mass (ASM), ASM index (ASM/height²), fat mass (FM), %FM, visceral fat area, and femoral neck and lumbar spine bone mineral density and T-scores. In one participant with unilateral limb damage that precluded a reliable appendicular lean mass (ASM) estimate for one limb, we estimated the missing segment using the lean mass of the contralateral (unaffected) limb to approximate the total contribution of that segment. This pragmatic approach was adopted to avoid systematic underestimation of ASM (and ASM/height²), which would occur if the missing or invalid limb were treated as zero and could lead to potential misclassification of sarcopenia status. The usefulness of DEXA in unilateral amputees has been acknowledged because it provides detailed regional body composition data that allow comparison between left and right body segments; thus, estimates of the missing extremity can be made compared with the intact side (19). For the purpose of confirmed sarcopenia diagnosis, the cutoff for ASM index was < 7.0 kg/m² (men) or < 5.5 kg/m² (women) (10). Adjusting ASM for stature reduces the influence of body size and minimizes potential misclassification when applying cutoff points derived from populations with different anthropometric characteristics. Therefore, the use of ASM normalized to height squared provides a more standardized and comparable metric across populations. Assessments for osteopenia and osteoporosis were conducted according to Bone Health and Osteoporosis Foundation (BHOF) guidelines (20). DEXA was performed using a Lunar Prodigy Advance system (GE Healthcare) with enCORE software, version 17.

The Short Physical Performance Battery (SPPB) was applied to assess physical performance. This test is composed of three domains: standing balance, 4-m gait speed, and the FTSTS test. Since each domain is scored from 0 to 4, a score < 4 indicates that the participant did not achieve the highest performance level in that specific component. The overall SPPB score was calculated, for which a score < 8 points indicates inadequate physical performance, and the highest score is 12 (10). Gait speed ≤0.8 m/s also indicated low physical performance. The calf circumference (CC) was measured at the right calf’s greatest girth. It has recently been demonstrated that CC measured on either side, regardless of hand dominance, can be used as an appropriate method for screening sarcopenia. Jeong et al. (21) found that both right-handed and left-handed individuals showed a trend toward smaller CC on the left side, with this difference reaching statistical significance among right-handed participants. Based on these findings, we decided to adopt right-sided CC measurements for standardization purposes (21). Also, waist-to-hip ratio (WHR) was measured at standard landmarks (22). Body mass index (BMI) was calculated and expressed as kg/m². For BMI-based categorical classification, WHO cutoff points were applied for participants < 60 years, with overweight and obesity grouped into a single “overweight” category (underweight: BMI < 18.5 kg/m²; eutrophy: BMI ≥ 18.5 and ≤ 24.9 kg/m²; overweight: BMI ≥ 25.0 kg/m²) (23). For participants ≥ 60 years, age-specific cutoff points were used (underweight: BMI < 22.0 kg/m²; eutrophy: BMI ≥ 22.0 and ≤ 27.0 kg/m²; overweight: BMI > 27.0 kg/m²) (24). A retrospective 24-hour dietary recall was administered by a trained dietitian to estimate protein intake, which was calculated in g/kg/day using the Dietbox^®^ Nutrition Software.

Sarcopenia was classified as probable (low strength), confirmed (low strength + low muscle mass) and severe (confirmed + low physical performance) (10). In addition to categorical classification, muscle mass and handgrip strength were analyzed as continuous variables using quantile regression models, allowing detection of subtle differences beyond sarcopenia prevalence.

To optimize patient flow and minimize waiting time, assessments were organized into four rotating stations, allowing participants to move sequentially between stations rather than remaining idle while awaiting a fixed assessment order.

One station (duplicated and conducted by two geriatricians specialized in Parkinson’s disease and parkinsonian syndromes) included the medical evaluation, medical record review, medication assessment, clinical interview focused on parkinsonian symptoms, and administration of the MDS-UPDRS part III.

A second station consisted of physical performance assessments and sarcopenia-related components, conducted by trained physical education professionals.

A third station was dedicated to cognitive and depressive symptom assessments, including the MMSE and the GDS-15, conducted by trained medical students under supervision.

The fourth station involved body composition assessment (including DEXA), anthropometric measurements, and dietary intake evaluation, including the 24-hour dietary recall, and was conducted by two trained nutritionists.

Although the starting station varied among participants, all individuals completed the same assessments in a rotating manner, ensuring standardized evaluation while optimizing time efficiency and reducing fatigue.

Patients attending this outpatient service often undergo multidisciplinary assessments and administrative procedures during routine visits, which may require extended time at the facility. To minimize fatigue and improve participant comfort, continuous access to seating, restroom facilities, light snacks, and interaction with accompanying persons and other patients was provided. Assessments were conducted flexibly, allowing participants to pause, rest, or relax whenever needed. If fatigue persisted or if the participant preferred, evaluations could be interrupted and rescheduled for another day.

All assessments were performed during the “on” state to minimize the influence of parkinsonian motor symptoms on strength, balance, and gait performance measurements. Conducting the evaluations during the “off” state could have artificially underestimated physical performance due to transient motor fluctuations rather than reflecting underlying muscle function. Evaluations were performed within three hours of the previous dose of antiparkinsonian medication. If clinical signs of wearing-off were observed earlier, medication was administered before the assessments.

### Statistical analysis

Descriptive statistics were presented as number (percentage) for categorical variables and as means ± SD or medians (IQR) for quantitative variables. Bivariate analyses for categorical variables were performed using the chi-squared test and Fisher’s exact test. The Student’s t test and Mann-Whitney U test were used for continuous variables. Data normality was assessed through the Shapiro-Wilk test.

As patients in the control group were older and had higher prevalences of hypertension, diabetes and dyslipidemia, we conducted adjusted analyses:

- Logistic regression for binary outcomes, checking VIF for multicollinearity. SARC-F and SPPB domains were dichotomized: SARC-F domains > 0 considered altered; SPPB domain < 4 considered altered.
- Poisson regression for medication count.
- Multinomial regression for bone health status (normal, osteopenia and osteoporosis).
- Linear regression for BMI and CC (normal residuals).
- Quantile regression for other continuous variables.

The selection of these covariates for inclusion in the adjusted models was based on their well-established association with increased risk of sarcopenia, in order to avoid confounding effects when comparing sarcopenia-related parameters between PD patients and controls.

Exploratory analyses with Spearman’s correlation were performed to evaluate associations between total SARC-F and SPPB scores, strength, and muscle mass. JAMOVI 2.4.8 (The jamovi project) and STATA 18.0 (StataCorp LLC, College Station, TX, USA) were used for the statistical analysis.

## Results

### Demographic characteristics and clinical background

The study population comprised 250 patients (124 with PD), of whom one hundred and nine (43.6%) were female. The control group had a higher median age: 71 *vs*. 65.9 years. Higher prevalences of clinical conditions related to cardiovascular disease – hypertension, type 2 diabetes, dyslipidemia, and coronary artery disease – were observed among non-PD patients. Comparative analyses were therefore adjusted for age and the first three variables using multivariate analysis. **Table 1** shows the main demographic and clinical features. As would be expected, we found higher prevalence of sleep complaints and clinical manifestations related to dysautonomia. Gait and mobility issues were also more frequent – use of walking aids, falls in the past month and six months, and recurrent falls – and remained significant after adjustment. A greater proportion of PD patients reported physical activity ≥3 times/week, though this was not significant after adjustment. In both groups, most reported cognitive complaints and the median number of medications was ≥5. Specific PD clinical features are presented in **Table 2**.

**Table 1:** Sociodemographic and clinical characteristics of PD patients and controls: bivariate and adjusted analyses.

Variable	PD group (N=124)	Control group (N=126)	p	OR (95% CI)	p-adj	aOR (95% CI)
Gender			0.786	0.9 (0.5-1.5)	n/a	n/a
Female	53 (42.7%)	56 (44.4%)				
Male	71 (57.3%)	70 (55.6%)				
Age	65.9 (58.3 - 74.7)	71 (66 - 77)	**< 0.001**	n/a	n/a	n/a
BMI classification (n=244)						
Underweight	14 (11.8%)	9 (7.2%)	0.46	n/a	n/a	n/a
Eutrophy	39 (32.8%)	45 (36%)				
Overweight	66 (55.5%)	71 (56.8%)				
Hypertension	59 (47.6%)	94 (74.6%)	**<0.001**	0.3 (0.1-0.5)	n/a	n/a
Type 2 DM	14 (11.3%)	48 (38.1%)	**<0.001**	0.2 (0.1-0.4)	n/a	n/a
Dyslipidemia	21 (16.9%)	64 (50.8%)	**<0.001**	0.2 (0.1-0.3)	n/a	n/a
Previous stroke	5 (4%)	10 (7.9%)	0.194	0.5 (0.1-1.6)	n/a	n/a
Coronary artery disease	3 (2.4%)	12 (9.5%)	**0.018**	0.2 (0.06-0.9)	n/a	n/a
Alcohol use	15 (12.1%)	13 (10.5%)	0.688	1.1 (0.5-2.5)	0.592	1.2 (0.5-3.1)
Current cigarette smoking	3 (2.4%)	5 (4%)	0.72	0.5 (0.1-2.5)	0.158	0.3 (0.06-1.5)
Use of walking aid devices	25 (20.2%)	12 (9.7%)	**0.021**	2.3 (1.1-4.9)	**0.008**	3.1 (1.3-7.5)
Sleep complaints	105 (84.7%)	65 (54.2%)	**<0.001**	4.6 (2.5-8.5)	**<0.001**	4.7 (2.3-9.2)
Regular physical activity	39 (31.5%)	25 (19.8%)	**0.035**	1.8 (1.04-3.3)	0.504	1.2 (0.6-2.3)
Cognitive complaints	72 (59%)	68 (55.3%)	0.555	1.1 (0.7-1.9)	0.356	1.3 (0.7-2.3)
Urinary incontinence	58 (46.8%)	39 (31%)	**0.01**	1.9 (1.1-3.2)	**0.006**	2.3 (1.2-4.1)
Orthostatic hypotension	27 (21.8%)	3 (2.4%)	**<0.001**	11.4 (3.3-38.7)	**<0.001**	10.1 (2.7-37)
Erectile dysfunction	13 (10.5%)	3 (2.4%)	**0.009**	4.8 (1.3-17.3)	**0.02**	5.7 (1.3-25.1)
Constipation	56 (45.2%)	15 (11.9%)	**<0.001**	6.1 (3.2-11.6)	**<0.001**	7.5 (3.5-16)
Falls in the last month (n=248)	24 (19.4%)	11 (8.9%)	**0.018**	2.4 (1.1-5.2)	**0.023**	2.8 (1.1-6.8)
Falls in the last 6 months (n=247)	51 (41.1%)	28 (22.8%)	**0.002**	2.3 (1.3-4.1)	**0.003**	2.6 (1.4-5)
Recurrent falls (n=247)	33 (26.6%)	12 (9.8%)	**<0.001**	3.3 (1.6-6.8)	**0.002**	3.6 (1.5-8.3)
Number of medications	5 (4 – 7)	6 (3 – 7)	0.778	n/a	0.11	RR (95% CI)
						1.2 (0.9-1.4)

N (%); median (IQR); Unadjusted comparisons: Mann–Whitney U for age and number of medications; Chi-square for categorical variables, except current cigarette smoking, which used Fisher’s exact test. Adjusted analyses: logistic regression for categorical variables (aOR [95% CI] and p-adj), and Poisson regression for number of medications, reported as RR [95% CI]. PD, Parkinson’s disease; Type 2 DM, type 2 diabetes mellitus; OR (95% CI), odds ratio and 95% confidence interval; aOR (95% CI), adjusted odds ratio from logistic regression; RR (95% CI), risk ratio from Poisson regression; p-adj, p value from the adjusted model. N/a: not applicable. Bold value indicates p<0.0.5.

**Table 2:** Clinical characteristics specific to Parkinson’s disease patients.

Variable	Value
Time of disease (years)	9 (6 – 12.3)
Hoehn and Yahr Scale	
Stage 1	2 (1.6%)
Stage 1.5	1 (0.8%)
Stage 2	24 (19.4%)
Stage 2.5	53 (42.7%)
Stage 3	44 (35.5%)
MDS-UPDRS Part III score	43.5 (32.8-57.3)
UPDRS 3.9 Standing from a chair	1 (0-1)
UPDRS 3.10 Gait	2 (1-2)
UPDRS 3.11 Freezing of Gait	0 (0-0)
Score 0	97 (78.2%)
Score 1	22 (17.7%)
Score 2	5 (4.0%)
UPDRS 3.12 Postural stability	1 (1-2.25)
UPDRS 3.13 Posture	1 (1-3)
SE ADL	90 (80 – 90)
Dyskinesias	67 (54%)
Motor fluctuations	80 (64.5%)
Freezing of gait	53 (42.7%)
Hallucinations	26 (21%)
REM Sleep Behavior Disorder	69 (55.6%)
Levodopa Equivalent Dose (mg)	1140.7 ± 571.5

N (%); median (IQR). *Hoehn and Yahr* scale is reported as distribution across stages 1–3. *MDS-UPDRS Part III score* (Movement Disorders Society–Unified Parkinson’s Disease Rating Scale, part III) and its subitems are expressed as median (IQR), with categorical distributions shown where applicable. *SE-ADL* denotes Schwab and England Activities of Daily Living scale, expressed as median (IQR). *Levodopa equivalent dose* is presented as mean ± SD.

### Sarcopenia, physical performance, depression and cognition

Comparisons for sarcopenia, physical performance, depressive symptoms and cognitive status are displayed in **Table 3**. PD patients scored higher on SARC-F and across domains, with higher prevalence of positive screening. Although median handgrip strength was higher in PD in bivariate analysis, the adjusted analysis showed a non-significant trend toward lower strength. Regarding physical performance, patients in the control group had better results in the balance test, whereas PD patients performed better in the chair stand test. The prevalence of probable, confirmed and severe sarcopenia, as well as of osteopenia and osteoporosis, were similar between groups. Cognitive performance and depression also did not differ significantly. **Table 4** displays a comparison of sarcopenia parameters between PD and control groups stratified by age group. Similar proportional differences in positive SARC-F screening were observed in both age groups (40–60 years and >60 years), reaching statistical significance in the latter, likely due to the larger number of patients in this subgroup.

**Table 3:** Functional, sarcopenia-related, bone health, and neuropsychiatric characteristics of PD patients and controls: unadjusted and adjusted analyses.

Variable	PD group (N=124)	Control group (N=126)	p	OR(95% CI)	p-adj	Adjusted OR (CI 95%)
SARC-F domains						
Strength	73 (58.9%)	49 (38.9%)	**0.002**	2.2 (1.3-3.7)	**0.001**	2.6 (1.4-4.7)
Assistance in walking	56 (45.2%)	22 (17.5%)	**<0.001**	3.9 (2.1-6.9)	**<0.001**	4.9 (2.5-9.8)
Rise from a chair	87 (70.2%)	48 (38.1%)	**<0.001**	3.8 (2.2-6.4)	**<0.001**	4.1 (2.2-7.4)
Climb stairs	84 (67.7%)	76 (60.3%)	0.221	1.3 (0.8-2.3)	0.06	1.7 (0.9-3.2)
Falls	59 (47.6%)	43 (34.1%)	**0.03**	1.7 (1.05-2.9)	**0.038**	1.8 (1.03-3.3)
Positive SARC-F screening	61 (49.2%)	36 (28.8%)	**<0.001**	2.4 (1.4-4.0)	**<0.001**	2.7 (1.5-5.0)
SPPB domains						
Balance test score	47 (37.9%)	44 (34.9%)	0.624	1.1 (0.6-1.9)	**0.014**	2.2 (1.1-4.2)
Gait speed score	22 (18%)	40 (31.7%)	**0.01**	0.4 (0.2-0.8)	0.601	0.8 (0.4-1.6)
Chair stand test score	96 (77.4%)	121 (96%)	**<0.001**	0.1 (0.05-0.3)	**0.001**	0.1 (0.06-0.5)
SPPB total score <=8	44 (35.5%)	55 (43.7%)	0.187	0.7 (0.4-1.1)	0.367	1.3 (0.7-2.4)
Probable sarcopenia	25 (20.2%)	36 (28.6%)	0.122	0.6 (0.3-1.1)	0.802	1.09 (0.5-2.2)
Confirmed sarcopenia	12 (9.7%)	14 (11.1%)	0.71	0.8 (0.3-1.9)	0.713	1.2 (0.4-3.0)
Severe sarcopenia	4 (3.2%)	11 (8.7%)	0.067	0.3 (0.1-1.1)	0.36	0.5 (0.1-1.9)
Bone Health Status (BHOF) (n=242)			0.699	n/a		
Normal	36 (29.8%)	42 (34.7%)			n/a	n/a
Osteopenia	55 (45.5%)	50 (41.3%)			0.134	1.7 (0.8-3.4)
Osteoporosis	30 (24.8%)	29 (24%)			0.185	1.7 (0.7-3.8)
GDS score >= 5	62 (50.4%)	54 (42.9%)	0.232	1.3 (0.8-2.2)	0.177	1.4 (0.8-2.6)
Depression (DSM-V)	39 (31.5%)	40 (32.5%)	0.857	0.9 (0.5-1.6)	0.938	1.02 (0.5-1.8)
						β Coefficient (95% CI)
Total SARC-F score	4 (2 – 6)	2 (1 – 4)	<0.001	n/a	**<0.001**	2.0 (0.9 to 3.0)
Total SPPB score	9 (8 – 11)	9 (7.75 – 10)	0.15	n/a	0.548	-0.2 (-0.8 to 0.4)
Gait speed	1.38 (0.98 – 1.7)	0.97 (0.78 – 1.12)	<0.001	n/a	**<0.001**	0.37 (0.2 to 0.5)
Handgrip strength	28 (20 – 36)	24 (18 – 35)	0.28	n/a	0.076	-3 (-6.4 to 0.3)
GDS-15 (total score)	5 (2 - 7)	4 (2 - 6.75)	0.349	n/a	0.115	1.0 (-0.2 to 2.2)
MMSE (total score)	26 (22 - 27)	24 (20-27)	0.091	n/a	0.63	- 0.4 (-2.0 to 1.2)

Values are n (%) or median (IQR). Unadjusted comparisons: Mann–Whitney U for continuous variables and Chi-square for categorical variables; *n/a = not applicable*. Adjusted analyses: logistic regression for categorical variables (reported as adjusted OR [95% CI] and *p-adj*), and quantile regression for continuous variables (reported as β coefficient [95% CI]). PD, Parkinson’s disease; OR, odds ratio; aOR, adjusted odds ratio; CI, confidence interval; β, regression coefficient from quantile regression; SARC-F, Strength, Assistance with walking, Rising from a chair, Climbing stairs, Falls questionnaire; SPPB, Short Physical Performance Battery; BHOF, Bone Health Osteoporosis Foundation classification; GDS, Geriatric Depression Scale; DSM-V, Diagnostic and Statistical Manual of Mental Disorders, 5th edition; MMSE, Mini-Mental State Examination. Bold value indicates p<0.05.

**Table 4:** Comparison of sarcopenia parameters between PD and control groups stratified by age group.

Variable	40 – 60 years (N = 53)			> 60 years (N = 197)		
	PD group	Control group	p	PD group	Control group	p
Positive SARC-F screening	21 (51.2%)	3 (25%)	0.109	40 (48.2%)	33 (29.2%)	**0.007**
SPPB total score <=8	8 (18.5%)	2 (16.7%)	1.00*	36 (43.4%)	53 (46.5%)	0.664
Probable sarcopenia	4 (9.8%)	1 (8.3%)	1.00*	21 (25.3%)	35 (30.7%)	0.407
Confirmed sarcopenia	1 (2.4%)	0 (0)	1.00*	11 (13.3%)	14 (12.3%)	0.840
Severe sarcopenia	1 (2.4%)	0 (0)	1.00*	3 (3.6%)	11 (9.6%)	0.104

Values are presented as n (%). PD, Parkinson’s disease. Between-group comparisons were performed using the Chi-square test, except when indicated by an asterisk (*), which denotes the use of Fisher’s exact test.

Bold value indicates p<0.05.

Overall, we found in the exploratory analyses that SARC-F is more strongly correlated with physical performance than muscle mass or strength: scores were negatively correlated with handgrip strength (rho = −0.368, p < 0.001), ASM/Ht² (rho = −0.169, p = 0.007), SPPB score (rho = −0.529, p < 0.001), and gait speed (rho = −0.306, p < 0.001). Among PD patients, negative correlation with handgrip strength (rho = −0.325, p < 0.001), SPPB score (rho = −0.562, p < 0.001), and gait speed (rho = −0.452, p < 0.001) was found, but the ASM/Ht² correlation was not statistically significant (rho = −0.114, p = 0.20). Handgrip strength (rho = −0.500, p < 0.001), ASM/Ht² (rho = −0.238, p = 0.007), SPPB score (rho = −0.611, p < 0.001), and gait speed (rho = −0.524, p < 0.001) were all negatively correlated with SARC-F scores in the control group.

All patients in the PD group were evaluated during the “on” state to minimize the influence of parkinsonian motor symptoms on strength, balance, and gait performance measurements. Conducting the evaluations during the “off” state could have artificially underestimated physical performance due to transient motor fluctuations rather than reflecting underlying muscle function.

### Anthropometric and body composition

Group comparisons for anthropometric and body composition features are shown in **Table 5**. PD patients had lower calf circumference (p < 0.001) and lower fat mass with marginal statistical significance in adjusted analysis. Protein intake was also higher in this group.

**Table 5:** Comparison of anthropometric and body composition measures between PD patients and controls.

Variable	Total (N=250)	PD group (N=124)	Control group (N=126)	p	p-adj	β coefficient (95% CI)
BMI	27.1 +- 4.4	26.5 +-4.4	27.7 +- 4.26	**0.029**	0.112	-0.98 (-2.1 to 0.23)
Calf circumference	34.4 +- 3.9	33.7 +- 3.6	35.1 +- 4.2	**0.005**	**<0.001**	-2.0 (-3.1 to -0.9)
Waist-hip ratio	0.9 (0.85 – 0.95)	0.89 (0.84 – 0.94)	0.91 (0.86 – 0.95)	0.164	0.739	-0.004 (-0.03 to 0.02)
Visceral fat area (cm²)	121.6 (83.4–158.4)	115.4 (71.9–153.1)	127.4 (91–160.4)	**0.01**	0.608	-5.6 (-27.2 to 15.9)
Fat mass (%)	34.7 (27.7 – 40.4)	33.4 (25.6 – 39.6)	35.7 (29.8 – 41.2)	**0.007**	0.776	-0.4 (-3.4 to 2.5)
Fat mass (kg)	23.2 (16.9–27.8)	22.4 (14.4–27.5)	24.7 (19–28.2)	**0.01**	0.055	-2.7 (-5.6 to 0.06)
Lean mass (kg)	41.8 (34.4 – 49.4)	41.8 (36.0 – 49.1)	41.8 (33.3 – 50.0)	0.553	0.137	-2.8 (-6.4 to 0.9)
ASM (kg)	17.9 (14.4–22)	17.8 (15.1–22)	18 (13.8–22.8)	0.674	0.238	-1.1 (-3.1 to 0.7)
ASM/Ht² (kg/m²)	7.29 (6.44 – 8.25)	7.29 (6.47 – 8.16)	7.30 (6.40 – 8.25)	0.744	0.311	-0.2 (-0.6 to 0.2)
Femoral neck BMD (g/cm²)	0.871 (0.784 – 0.991)	0.883 (0.802 – 1.01)	0.863 (0.768 – 0.970)	0.278	0.47	-0.02 (-0.07 to 0.03)
Femoral neck T-score	-1.2 (-1.8 to -0.3)	-1.1 (-1.7 to -0.2)	-1.25 (-1.92 to -0.5)	0.263	0.38	-0.2 (-0.5 to 0.2)
Lumbar spine BMD (g/m²)	1.03 (0.899 – 1.16)	1.02 (0.888 – 1.16)	1.03 (0.905 – 1.16)	0.641	0.11	-0.05 (-0.1 to 0.1)
Lumbar spine T-score	-1.3 (-2.4 to -0.2)	-1.35 (-2.5 to -0.2)	-1.25 (-2.27 to -0.2)	0.573	0.28	-0.3 (-0.8 to 0.2)
Protein ingestion (g/kg/day)	1.03 (0.82–1.32)	1.15 (0.91–1.33)	0.93 (0.77–1.24)	<0.001	**0.03**	0.13 (0.01 to 1.9)

Values are expressed as mean ± SD or median (IQR), as appropriate. Unadjusted comparisons were performed using the Mann–Whitney U test or Student’s t test for continuous variables. Adjusted analyses used quantile or linear regression, reported as β coefficients (95% CI) with adjusted *p* values. PD, Parkinson’s disease; BMI, body mass index; ASM, appendicular skeletal muscle mass; ASM/Ht², appendicular skeletal muscle mass index (kg/m²); BMD, bone mineral density; p-adj, p value from the adjusted model. Bold value indicates p<0.05.

**Figure 1:**
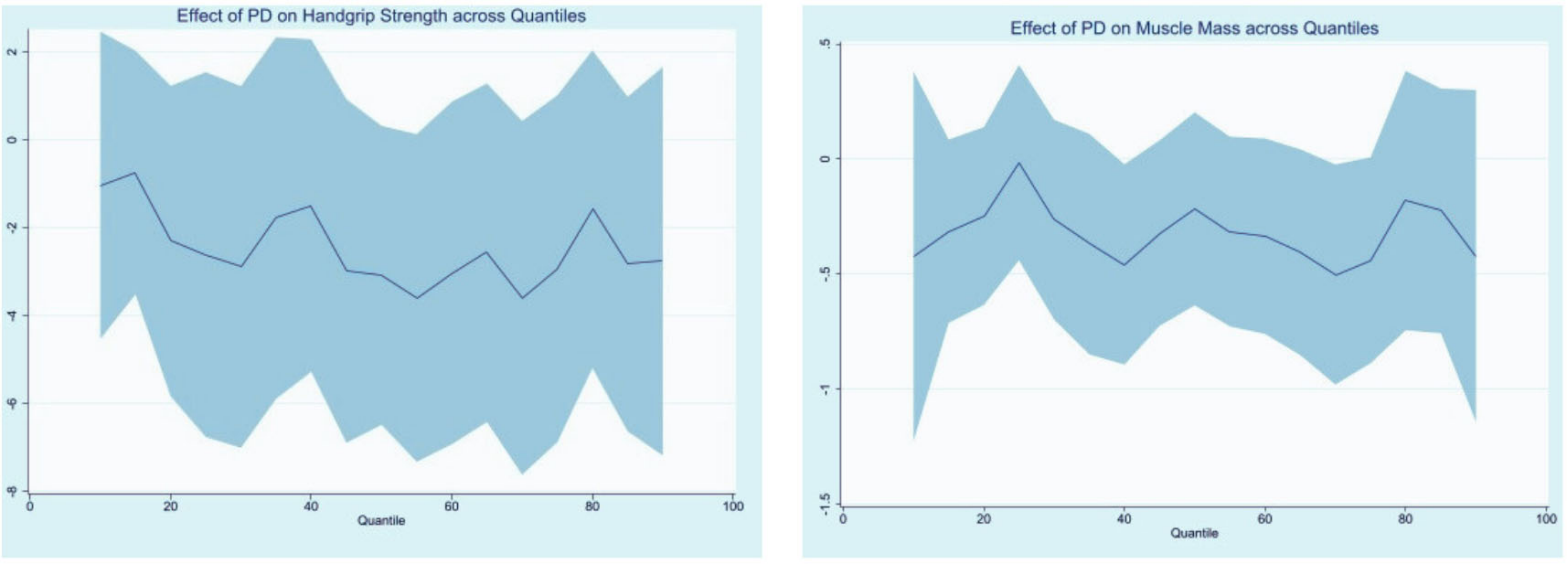
Quantile regression of handgrip strength (kg) and appendicular skeletal muscle mass index (kg/m2) across quantiles (x- axis) in Parkinson's disease patients versus adjusted controls. Quantile regression models were fitted for each measure, with the quantiles represented on the x-axis and the estimated values on the y-axis. The left panel shows handgrip strength (kg), and the right panel depicts appendicular skeletal muscle mass index (kg/m2). PD patients are compared with controls matched for age and adjusted for hypertension, type 2 diabetes, and dyslipidemia. The shaded areas represent 95% confidence intervals. PD: Parkinson's disease.

**Figure 1** illustrates associations between PD status and both handgrip strength and appendicular skeletal muscle mass index across quantiles, adjusted for covariates. We observed a trend toward lower values for both muscle mass and strength, with a significant reduction in muscle mass at the 40th percentile (p=0.039; β −0.45, 95% CI −0.89 to −0.02).

## Discussion

The main objective of this study was to assess sarcopenia parameters of middle-aged and older patients with mild to moderate PD in comparison with a matched group without PD. All analyses were adjusted for age, hypertension, type 2 diabetes, and dyslipidemia, which are well-established risk factors for sarcopenia that differed between groups (10, 25–27). Although sarcopenia prevalence did not differ significantly, PD patients scored higher on the SARC-F overall and across most domains, performed worse on SPPB balance, and showed a trend toward lower handgrip strength and appendicular skeletal muscle mass index across quantiles, with a significant reduction in muscle mass at the 40^th^ percentile. Also, sleep complaints and clinical features related to dysautonomia were more frequent in this group, with an increased frequency of falls.

Contrary to previous studies (13, 28, 29), we did not observe higher sarcopenia prevalence among PD patients. Estimating the prevalence of sarcopenia in patients with PD has been challenging, and several factors may explain discrepancies across studies. Definitions and diagnostic criteria vary, with region-specific cut-offs and no global consensus (8, 9, 18, 28–34). The recently established Global Leadership Initiative in Sarcopenia (GLIS) may advance a universal framework (35). Second, patient characteristics differ: our study did not include advanced cases, where sarcopenia is expected to be more prevalent. Also, relatively higher levodopa equivalent doses may have helped preserve muscle mass and strength (36, 37). Improvements in distal muscle activation (37) and in bradykinetic and hypometric spatial components of gait and turning have been reported (38). Higher levodopa intake has been linked to increased ASM index and handgrip strength (30). It also influences force development, and discontinuation reduces strength (39, 40).

Since 2019, the Movement Disorders outpatient clinic at HUWC has included a geriatrician and a team of medical residents in Geriatrics. These professionals have been providing proper guidance to patients regarding adequate protein intake. Recent study conducted by our group found that low protein intake (< 1 g/kg/day) was associated with positive screening of sarcopenia (SARC-F ≥ 4), low ASM, and high fat mass index in PD patients (41). This outpatient clinic has served as a setting for clinical research involving interventions such as the PARK-BAND Study, which included a health education program consisting of regular meetings to discuss educational topics on PD (42). The program was supported by a 12-chapter booklet containing informative content on clinical features and treatment strategies, including regular physical exercise and adequate protein consumption. We believe that these interventions likely contributed to the recent findings of higher protein intake in this sample and a lower prevalence of sarcopenia than would be expected based on previous studies.

Despite these factors, PD patients showed a trend toward 3 kg lower handgrip strength, with consistent declines across quantiles. Although not statistically significant (p=0.076), this pattern suggests a potential association with reduced muscle strength. A similar downward trend was seen for muscle mass, with significant reduction at the 40^th^ percentile (p=0.039). These findings support a possible adverse association between PD and muscle strength and mass, even at mild-to-moderate stages and despite optimized treatment and protein intake. The downward shift across the distribution suggests a potential reduction in muscle strength and mass not fully captured by mean comparisons, as group differences may be more relevant in specific segments of the distribution. These results illustrate the added value of quantile regression in revealing clinically meaningful PD-related differences that may disproportionately affect specific segments of the population and would remain undetected in mean-based analyses. The absence of significant differences in sarcopenia prevalence between groups should not be interpreted as evidence of preserved muscle health in PD. Rather, it may reflect limitations of current diagnostic criteria, which rely on fixed cutoff points and may lack sensitivity to detect early and subtle impairments, particularly in disease-specific contexts.

PD patients had a higher frequency of falls and recurrent falls. A recent study conducted by our group also demonstrated that several variables related to disease progression, treatment, and motor complications – such as hallucinations, dyskinesias, and the use of amantadine and COMT inhibitors – may contribute to falls in this population (43). The present study further contributes to the current knowledge by demonstrating worse balance compared to matched non-PD individuals. Our findings suggest that, despite its already well acknowledged limitations in detecting sarcopenia in PD patients, the SARC-F may serve as a useful tool for identifying individuals with balance and mobility impairments, including those at increased fall risk, an area that warrants further investigation in prospective studies. Considering the overall evidence regarding its feasibility and cost-effectiveness, it seems reasonable to continue using the SARC-F in clinical practice, particularly in fast-paced settings.

The present study, which included PD patients during the “on” phase, found that altered chair stand test was less frequent in this group compared to controls. Also, PD patients showed higher gait speed. Recent study by Baudendistel et al. (44)demonstrated that, PD patients on medication exhibited increased gait speed, primarily due to improvements in step length and step time. While peak propulsive force and hip flexion torques increased with medication, hip extensor and ankle plantarflexor torques did not. This disproportionate recruitment of muscle force may represent a limiting factor for the effectiveness of levodopa as an intervention to improve walking (44). Also, levodopa may normalize gait measures to healthy control levels (45). In our study, fear of stigma might have increased participants’ motivation to perform better on particular speed-dependent tests. In this context, the combination of higher gait speed with impaired balance may have contributed to the higher prevalence of falls in PD patients. These findings underscore the complexity of clinical care in this population, where the motor benefits of antiparkinsonian medications must be carefully weighed against potential adverse effects associated with both treatment and disease progression. The higher prevalence of orthostatic hypotension may also contribute to an increased risk of falls, and, together with other manifestations of dysautonomia – such as constipation, urinary incontinence, and erectile dysfunction – further adds to the overall burden of the disease.

The present study found lower CC and lower fat mass among PD patients, which aligns with previous studies. PD patients have been consistently found to have lower BMI and fat mass than matched controls. Loss of body fat mass accounts for a significant portion of the overall weight loss observed in these patients. Song et al. (46) reported that declines in total body and fat mass started approximately 6–7 years before diagnosis, although reductions did not reach statistical significance until around 3–5 years following diagnosis, and 96% of the weight loss was due to fat mass reduction (46). Various factors contribute to these findings: reduced appetite due to altered olfaction and constipation may impact nutrition, and motor symptoms such as tremor, muscle rigidity, and dyskinesia may increase energy expenditure (3).

The present study has some limitations. This study was cross-sectional and therefore did not assess longitudinal changes in muscle mass, strength, or physical performance, nor did it evaluate intervention effects or long-term clinical outcomes. Although groups were matched by sex, potential sex-related differences in sarcopenia expression were not formally analyzed and may warrant further investigation in larger samples. Patients with advanced PD (Hoehn and Yahr stages 4–5) and those wheelchair-bound were not included. Therefore, the reported prevalence of sarcopenia and comparisons with controls may not be generalizable to individuals with advanced stages. Additionally, normative reference curves and specific cut-off points for muscle mass and strength in PD patients have not yet been established. Cut-off points applied in this study were those recommended by the EWGSOP2. Moreover, there are currently no methods specifically validated to diagnose sarcopenia by assessing lower limb muscle strength in PD patients. Finally, given the study setting – a tertiary university hospital – it was not possible to assemble a control group primarily matched for age and major cardiovascular comorbidities. Nevertheless, all statistical analyses were appropriately adjusted for these potential confounders to ensure a reliable comparative assessment reflective of our population.

This cross-sectional comparative study with matched controls revealed a trend toward lower handgrip strength and ASM index among mild-to-moderate PD patients across quantiles, with a significant reduction in muscle mass at the 40^th^ percentile, despite higher protein intake and similar prevalences of sarcopenia. These results highlight subtle but clinically relevant impairments that may not be captured by traditional mean-based analyses and categorical sarcopenia diagnosis. PD patients exhibited lower fat mass and CC. PD patients also demonstrated worse balance, higher gait speed and increased prevalence of falls, along with more complaints related to dysautonomia. SARC-F was a better indicator of physical performance than of muscle mass or strength. Optimized pharmacological treatment as well as proper nutritional and physical exercise interventions aimed at preserving muscle mass and function, improving balance and reducing the risk of falls should be integrated into the care of patients living with PD.

## Data Availability

The raw data supporting the conclusions of this article will be made available by the authors, without undue reservation.
